# The MATISSE study: a randomised trial of group art therapy for people with schizophrenia

**DOI:** 10.1186/1471-244X-10-65

**Published:** 2010-08-27

**Authors:** Mike J Crawford, Helen Killaspy, Eleftheria Kalaitzaki, Barbara Barrett, Sarah Byford, Sue Patterson, Tony Soteriou, Francis A O'Neill, Katie Clayton, Anna Maratos, Thomas R Barnes, David Osborn, Tony Johnson, Michael King, Peter Tyrer, Diana Waller

**Affiliations:** 1 Centre for Mental Health, Imperial College, Claybrook Road London, W6 8LN, UK; 2 Department of Mental Health Sciences, University College London, Pond Street, London, NW3 2QG, UK; 3 MRC General Practice Research Framework, North Gower Street, London, NW1 2ND, UK; 4 Centre for the Economics of Mental Health, King's College London, De Crespigny Park, London SE5 8AF, UK; 5 Avon and Wiltshire Mental Health Partnership NHS Trust, Jenner House,Langley Park, Chippenham, SN15 1GG, UK; 6 Centre for Public Health, Queen's University, Grosvenor Road, Belfast, BT12 6BA, UK; 7 Camden and Islington NHS Foundation Trust, St Pancras Way, London, NW1 OPE, UK; 8 Central and North West London NHS Foundation Trust, Hampstead Road, London, NW1 7QY, UK; 9 MRC Biostatistics Unit, Cambridge and MRC Clinical Trials Unit, Robinson Way, Cambridge, CB2 0SR, UK

## Abstract

**Background:**

Art Therapy has been promoted as a means of helping people who may find it difficult to express themselves verbally engage in psychological treatment. Group Art Therapy has been widely used as an adjunctive treatment for people with schizophrenia but there have been few attempts to examine its effects and cost effectiveness has not been examined. The MATISSE study aims to evaluate the clinical and cost effectiveness of group Art Therapy for people with schizophrenia.

**Method/Design:**

The MATISSE study is a three-arm, parallel group, pragmatic, randomised, controlled trial of referral to group Art Therapy plus standard care, referral to an attention control 'activity' group plus standard care, or standard care alone. Study participants were recruited from inpatient and community-based mental health and social care services at four centres in England and Northern Ireland. Participants were aged over 18 years with a clinical diagnosis of schizophrenia, confirmed by an examination of case notes using operationalised criteria. Participants were then randomised via an independent and remote telephone randomisation service using permuted stacked blocks, stratified by site. Art Therapy and activity groups were made available to participants once a week for up to 12 months. Outcome measures were assessed by researchers masked to allocation status at 12 and 24 months after randomisation. Participants and care givers were aware which arm of the trial participants were allocated to. The primary outcomes for the study are global functioning (measured using the Global Assessment of Functioning scale) and mental health symptoms (measured using the Positive and Negative Syndrome Scale) assessed at 24 months. Secondary outcomes were assessed at 12 and 24 months and comprise levels of group attendance, social function, satisfaction with care, mental wellbeing, and costs.

**Discussion:**

We believe that this is the first large scale pragmatic trial of Art Therapy for people with schizophrenia.

**Trial registration:**

Current Controlled Trials ISRCTN46150447

## Background

Schizophrenia is a severe mental disorder which affects as many as one in 100 people at some point in their lives [1]. In addition to 'positive' symptoms of schizophrenia such as hallucinations and delusions, many people also experience varying degrees of loss of energy, impaired attention, reductions in the amount and content of speech and other so-called 'negative' symptoms [2]. While antipsychotic medication reduces the symptoms of schizophrenia and decreases the likelihood of relapse [3], many people do not adhere to treatment and a substantial proportion of those who do experience residual symptoms, relapse and reduced social functioning [4,5]. Psychological and social interventions are widely used in combination with pharmacotherapy in an effort to further improve the health and social outcomes of people with schizophrenia and several have been shown to be effective [6].

Art Therapy is a form of psychotherapy that has been practised for over 60 years [7]. It has been promoted as a means of helping people who may find it difficult to express themselves verbally engage in psychological treatment. In Art Therapy people are provided with a choice of art materials and encouraged to use them to express themselves freely. It has been argued that Art Therapy has advantages over traditional psychotherapies because the images that a person makes can help a person understand themselves better whilst also containing powerful feelings that might otherwise overwhelm them [8]. The key ingredients of Art Therapy are considered to be the process of making art, and the relationship that develops between the therapist and the participant [9]. In group Art Therapy, there is also the potential to explore and utilise the experience of other relationships between group members [10].

Despite the widespread use of group Art Therapy for people with schizophrenia little research has been conducted to explore its effects [11]. Green and colleagues conducted a randomised trial of 10 weekly sessions of group Art Therapy plus standard care versus standard care alone among 47 people with 'chronic psychiatric disorders' of whom half had a clinical diagnosis of schizophrenia [12]. At 10-week follow-up those allocated to group Art Therapy reported improved self esteem. More recently, Meng and colleagues randomised 86 in-patients to twice weekly group Art Therapy delivered over 15 weeks and reported improved health and social functioning at the end of this period [13]. Richardson and colleagues [14] compared the addition of 12 weekly sessions of group Art Therapy to standard care among people with chronic schizophrenia being treated in outpatient settings. Among 40 (45%) participants who were followed up at six months, statistically significant reductions in negative symptoms were found.

However, in their systematic review of the effectiveness of Art Therapy for people with schizophrenia, Ruddy and Milnes [15] concluded that because of small sample sizes, short follow-up periods, and high rates of loss to follow-up, the benefits and potential harms of Art Therapy for people with schizophrenia are still unclear. Moreover because previous studies have not incorporated attention control groups there is no evidence regarding the relative contribution of non-specific components and 'active ingredients' of the intervention to observed outcomes. Nor has previous research examined the costs or cost effectiveness of this intervention.

### Research objectives

The objectives of the study are to examine the impact of referral to group Art Therapy plus standard care in people with schizophrenia compared to referral to attention control treatment plus standard care or standard care alone on health and social functioning and to compare the costs and cost effectiveness of adding group Art Therapy to a person's existing treatment.

The study hypotheses are that, among people with schizophrenia;

i) Referral to group Art Therapy is associated with improved global functioning at 24 months compared to referral to attention control treatment or standard care alone.

ii) Referral to group Art Therapy is more cost-effective than referral to attention control treatment or standard care alone.

iii) Referral for group Art Therapy is associated with improved mental health, social functioning, well-being and satisfaction with care compared to referral for attention control treatment or standard care alone.

iv) Those referred to group Art Therapy will attend a greater proportion of the groups available to them than those referred to activity groups.

Our primary hypothesis is based on global functioning and symptoms of psychosis at 24 months. We have selected this time point because previous studies of psychosocial interventions for people with schizophrenia have demonstrated greater improvements in global functioning in the period after the end of therapy [16,17].

## Methods

### Trial design

The MATISSE study (Multi-centre study of Art Therapy In Schizophrenia - Systematic Evaluation) is a three-arm, parallel group, pragmatic, randomised, controlled trial of referral to group Art Therapy plus standard care, referral to an attention control 'activity' group plus standard care, or standard care alone. Similar numbers of participants were randomised to each of the three arms of the trial. We aimed to use a pragmatic design which would allow us to test the impact of referring people to group Art Therapy in normal clinical practice.

Three changes were made to the design of the study after commencement. Firstly, because recruitment was slower than anticipated the period for recruiting the study sample was increased from nine to 20 months [18]. Secondly, following publication of national guidance on the treatment of schizophrenia highlighting the importance of arts therapies in treating symptoms of schizophrenia [19], we promoted total symptom score as a co-primary outcome measure. Finally, early data demonstrating lower levels of attendance at groups than we anticipated led us to increase the total number of participants to 10% above our original target. Ethical approval for the study, including these protocol amendments, was given by Huntingdon Research Ethics Committee (06/Q0104/82) and the study protocol was registered with Controlled Clinical Trials (ISRCTN46150447) prior to the start of data collection.

### Study setting and sample

Study participants were recruited from four UK centres, three in England (West London, North London, and Avon & Wiltshire) and one in Northern Ireland (Belfast). Centres were selected because they had systems for delivering group Art Therapy to people with schizophrenia and for supervising and supporting arts therapists. The centres cover a mix of inner city, urban, semi-rural and rural areas and serve a population that includes people from a variety of different ethnic backgrounds.

We recruited participants from secondary care settings including inpatient units, day hospitals, community mental health teams, rehabilitation services, supported accommodation and day centres. To take part in the study people had to be aged 18 years or over and have a clinical diagnosis of schizophrenia, confirmed by an examination of case notes using operationalised criteria (OPCRIT) [20]. Exclusion criteria were minimised to increase the generalisability of study findings. A list of all inclusion and exclusion criteria is presented in Table 1. While people who were currently receiving Art Therapy or another of the arts therapies (Music, Drama Therapy etc) were excluded from the study, those who were in receipt of other forms of structured psychosocial intervention were included.

**Table 1:** Inclusion and exclusion criteria for the MATISSE study

Inclusion criteria	Exclusion criteria
Aged 18 years or over	Already receiving Art Therapy or another arts therapy (Music Therapy, Drama Therapy, or Dance/Movement Therapy)
Clinical diagnosis of schizophrenia confirmed using operationalised criteria (OCRIT) [20]	Severe cognitive impairment
Willing to provide written informed consent. Willing to take part in trial therapies	Inability to speak sufficient English to complete the baseline assessment

#### Study interventions

The MATISSE trial has three treatment conditions: referral to group Art Therapy plus standard care, referral to an activity group plus standard care, and standard care alone. The guidance given to group facilitators on processes and response to adverse events of Art Therapy and activity groups used in the trial is summarised in Tables 2 and 3.

**Table 2:** Group processes and response to adverse events used in the trial

Aspect of structure or content of groups	Aspects shared by Art Therapy and activity groups
Engaging with the group	Group facilitators should contact new members by post and or telephone to invite them to the group and provide them with details of location, start times etc. Facilitators should try to meet participants on one occasion before they commence the group to outline aims, protocol boundaries and expectations. This may be done either individually or in groups

Group member leaves the group	When a group member specifically tells the facilitator that that they do not want to attend the group, or when they have not attended the group for a number of weeks without there being a clear reason for the facilitator should use their clinical judgement to make a decision about when they should be considered as having left the group. At this stage the facilitator will write to the patient confirming that their place in the group has closed

Replacing a group member with another patient	When it is agreed that a patient has left the group the facilitator should notify the trial coordinator who will make a note that there is a space in the group that can be filled by another study participant

Verbal aggression or violence	Facilitator to obtain and refer to risk assessment for all group members prior to their joining. In case of agitation/aggression/violence, the facilitator should use their clinical judgement to assess the situation and attempt de-escalation. The group member may need to be asked to leave the room. Inform the patient's care-coordinator, document the incident on the treatment fidelity proforma and complete incident form etc (as per usual clinical practice). Patients may be asked to stay away from subsequent groups (such a decision should be discussed with clinical supervisor)

Deteriorating mental state	Where a participant's mental state shows clear signs of deteriorating the facilitator should encourage the patient to discuss this with their care coordinator or psychiatrist. If the situation continues to deteriorate the facilitator should seek verbal consent from the patient to contact their care coordinator. In consultation with their supervisor and following review of their risk assessment and care plan, there may be circumstances in which the facilitator will need to contact the patient's care coordinator even if consent is withheld

Therapist leaves local services OR sick leave etc	When long gaps look likely the situation should be discussed with the local supervisor and efforts made to identify a new facilitator. Participants should be given as much notice of this as possible

**Table 3:** Differences in group processes and response to adverse events in Art Therapy and activity groups used in the trial

Aspect of structure or content	Activity Groups	Art Therapy groups
Late attendance	Remind client about starting times	Use clinical judgement when deciding how to explore reasons for late attendance/feelings about the group

Conflict with facilitator/therapist or other group members	Make efforts to help the patient calm themselves, try to refocus patient on group activities, and try to take steps to avoid escalation of the situation	Use clinical judgement to enquire about reasons for conflict and understand the behaviour in terms of their art work, group processes, and other factors in the patient's life

Annual leave/sick leave	MATISSE group supervisors should discuss this with individual group facilitators but we suggest that every attempt is made to avoid absence of facilitators during the first few weeks of the study. Once a group has become established short periods of leave should be managed by the co-facilitator	If the art therapist is unable to attend the group the group will be cancelled
		Wherever possible the group will be notified in advance and space provided for members to process this

Handling psychological material	If participants raise psychological concerns these should be handled in a sensitive, client-centred manner by the facilitator. Diversionary methods may be used to help participants focus on group activities as a means of distracting themselves from their symptoms. Participants may also be encouraged to raise their concerns with their key worker	Art therapists should use their clinical judgement to decide how to help participants express themselves both verbally and through use of images. Experiences of distress may be considered in the context of factors occurring in their lives and the outside world, but may also be thought about in relation to group processes and their use of art materials. While therapists may sometimes suggest links between art work and the persons' mental state or history, therapy is generally focussed on the 'here and now'. Efforts to address the content and meaning of art work produced by a person who is acutely psychotic need to be handled with utmost sensitivity or avoided
	Psychological concerns will not be explored in these groups and interpretations of participants' behaviours or comments must not be provided	

Group facilitator leaves	Changes in group facilitator should be explained ahead of any change wherever possible	Opportunities for exploring participants' feelings about changes of facilitator should be made available

#### Group Art Therapy

Those randomised to group Art Therapy were offered weekly sessions of 90 minutes of duration for a period of 12 months. We planned that no group would have more than eight 'active' members, though more than eight people were sometimes referred when those allocated did not engage (see table 2). All groups were led by art therapists registered with the Health Professions Council who had previous experience working with people with psychosis. Groups were co-facilitated by another member of staff or a volunteer.

Group Art Therapy was conducted in keeping with recommendations of the British Association of Art Therapists [21]. The key ingredients of group Art Therapy are considered to be the process of art making, and the tri-partite relationship which involves therapist, participant and image [9]. The groups aim to give people the potential to explore and utilise the experience of other relationships between group members [10]. A range of art materials was available in each group and participants were encouraged to use them to express themselves freely and spontaneously. Relationships within the group were considered in relation to both conscious and unconscious processes. Art therapists generally adopted a supportive approach, offering empathy and encouragement. They rarely provided symbolic interpretations of inter-personal process or images. They did however frequently discuss these processes in supervision. Within this framework, therapists employed a range of interventions thought appropriate to each participant. This approach is in keeping with recommendations for the pragmatic evaluation of complex interventions [22] in which individual therapists are encouraged to apply treatment principles flexibly to fit with the needs of participants [23].

#### Activity groups

Activity groups were designed to control for the non-specific effects of group Art Therapy; identified as structured time with an empathic professional and opportunities for interaction with peers in a group setting. They were also designed to reflect the kind of activity-based groups currently provided by mental health and social care services for people with psychosis in the UK. Allocated participants were offered a place in a weekly activity groups of for up to 90 minutes duration for a 12 month period. No group had more than eight members, though more than eight people could be referred to a group to support membership up to this level. All lead facilitators had previous experience of working with people with psychosis in groups and all groups were co-facilitated by another member of staff or volunteer.

Group facilitators offered various activities to members and encouraged participants to collectively select activities for the group. Activities included themed discussion, board games, watching and discussing DVDs, visits to local cafes and occasional visits to places of interest. The use of art and craft materials was prohibited. Group facilitators were asked not to engage participants in therapeutic conversation. Where necessary, if for example participants became distressed or wanted to discuss clinical concerns, facilitators employed diversion and/or encouraged participants to take up any specific concerns with professionals already involved in their care.

Prior to entry into groups art therapists and activity group facilitators met participants individually or in small groups to provide information about the group and promote engagement. Telephone and postal contact with participants and those involved in their care was used to promote engagement and retention in groups.

#### Standard care

Standard care involved follow-up from secondary care mental health services, care coordination, pharmacotherapy and the option of referral to other services. No restrictions were imposed on referral to other services apart from arts therapies which participants agreed not to use until the final follow-up assessment had been completed.

### Treatment fidelity

Facilitators of all Art Therapy and activity groups completed a short proforma at the end of each group. The form required the facilitator to note the structure and content of the group including: the names and number attending and duration of attendance, any breaches of group boundaries and how these were addressed, the verbal content of sessions and responses made by group facilitators to verbal content. For Art Therapy groups, therapists were also asked to record the art materials made available and used by the group, and for activity groups facilitators were asked to record the principal activities pursued.

All art therapists and facilitators of activity groups attended an orientation meeting at the start of the study. The background and methods of the project were presented and general principles for facilitating groups, arrangements for supervision, and the role of study proforma were discussed. During the treatment phase of the trial, art therapists and activity group facilitators received local monthly group supervision. Supervision sessions were audio-recorded and recordings reviewed by a senior member of the study team who provided feedback to supervisors regarding adherence to general guidelines as presented in Table 2.

At the end of the study, proforma from all centres were collected by the research team and a random sample of 50 (25 from Art Therapy groups and 25 from activity groups) per study centre (i.e. 200 in total) were examined for treatment fidelity. Data on *'verbal content of sessions and responses made by group facilitators' *were extracted. Specific references to the type of group were removed and a senior member of the study team, masked to what type of group the data was extracted from, rated each extract as coming from either an Art Therapy group or an activity group.

### Measures

At baseline, demographic and clinical data were collected including; age, gender, ethnicity, highest level of educational achievement, employment status, housing status, date of first presentation to clinical services with schizophrenia, primary and any secondary clinical diagnosis, current medication, and previous receipt of structured psychosocial interventions including arts therapies. Written records and in some cases collateral information gathered from carers or health professions were used to generate a psychiatric diagnosis using operationalised criteria [20]. Primary and secondary outcome measures are listed below. Each measure was assessed at recruitment (baseline), one year and two year follow-up. Measures were completed either by the researcher, the participant or by their key worker as indicated below.

#### Completed by the researcher

i) Global functioning (co-primary outcome) - was assessed using the Global Assessment of Functioning Scale (GAF), a 100-point single item, observer-rated scale that rates functioning on a continuum from health to illness. It is a reliable and valid measure of global functioning that has been widely used in previous studies of people with schizophrenia and is sensitive to change [24].

ii) Mental health (co-primary outcome) - was assessed using the Positive and Negative Syndrome Scale [25]. This is a 30-item rating scale which is accompanied by a structured interview. It takes approximately 30 minutes to complete and has been widely used to examine changes in symptoms in people with schizophrenia and related psychoses.

iii) Medication - was recorded all medication being prescribed to participants and assessed concordance using the Morisky Scale a four item questionnaire which provides a valid estimate of use of psychotropic medication [26].

iv) Health related quality of life - was assessed using Euroqol EQ-5 D [27]. This is a generic measure for describing and valuing health-related quality of life assessed in five domains (mobility, self-care, usual activities, pain/discomfort, anxiety/depression).

v) Cost data - was assessed using a modified version of the Adult Service Use Inventory which was designed on the basis of previous studies in adult mental health populations [28,29] and adapted for the purpose of this study.

#### Completed by the participant

vi) Social Function - was assessed using the Social Function Schedule [30], a widely used self-completed measure of social function with established reliability and validity.

vii) Wellbeing was assessed using the General Well-Being Scale. This 18 item, self-report instrument was originally developed for the US Health and Nutrition Survey, but has subsequently been used in studies of people with schizophrenia and has good psychometric properties [31].

viii) Satisfaction with mental health services - was assessed using the Client Satisfaction Questionnaire, an eight-item measure that has been widely used in previous studies and is sensitive to change [32].

#### Completed by the participants' key worker

ix) Engagement with mental health services was assessed using the four-item Service Engagement Scale [33].

x) Data on occupational and housing status were gathered indicating whether the participant lived in independent or supported accommodation (and the degree of support provided), together with a short description of any paid work, voluntary work or educational/training activities undertaken by the participant during the previous six months.

xi) Any incidents of suicidal behaviour, violence or aggression in the previous year were recorded using a proforma based on the one used by Johnson and colleagues [34].

xii) Global functioning using the Global Assessment of Functioning Scale (GAF) [24]

was rated by the researcher in instances where it was not possible for them to complete a face-to face assessment of the participant. A 'proxy GAF' based on best available information from whatever contact they had had with the participant, key informants and clinicians was made.

Following the collection of all 24-month follow-up data, participants' electronic and written records were examined to obtain details of any period of inpatient treatment received during the previous two years.

### Study procedures

In each centre researchers publicised the study through meetings with staff at local inpatient units, community teams, day centres and residential units. Researchers visited these teams on a regular basis to remind staff about the study and promote recruitment of potential participants. Researchers were assisted in this by clinical studies officers of the UK Mental Health Research Network. Clinical staff were given a copy of an information sheet which summarised the study protocol and helped them identify patients who may be suitable for the study. Researchers met those who had given verbal consent to be approached about the study, assessed eligibility, provided written and verbal information, obtained written consent, and collected baseline data. Participants were then randomised via an independent remote telephone randomisation service using permuted stacked blocks, stratified by site. The block size was randomly assigned between three and six. Each element within the block was randomly assigned to one of the three treatments in proportion to the size of the block.

Participants, their key worker and their general practitioner were notified of allocation status by an independent administrator. The administrator simultaneously informed local art therapists or activity group facilitators of the allocation status of the participant so that arrangements could be made for the participant to receive their allocated intervention while researchers involved in collecting follow-up data remained masked.

Rater 'masking' was maintained by providing specific instructions to participants and their clinical teams not to disclose treatment details. Data are held securely and all personal identifiers removed, with randomisation details held separately and password protected. Data on participants' uptake of the trial interventions was monitored through proforma completed by group facilitators after each group as described above. Thus researchers did not have to record this information from case files as this would have led to unmasking. Participants completing follow-up interviews were offered a £15 honorarium in recognition of their time in completing research interviews and any inconvenience related to their involvement in the study.

### Sample size

The sample size calculation for the study was based on the primary hypothesis: that those referred to group Art Therapy will have improved global functioning at 24 months compared to those referred to attention control treatment or standard care alone. Global functioning had not been assessed in randomised trials of Art Therapy for people with schizophrenia that had been completed when the study was being planned, so data on mean GAF scores and standard deviations were taken from previous trials of Compliance Therapy and Cognitive Therapy for people with schizophrenia. These interventions demonstrate an improvement in GAF scores of between five and 10 points [16,17]. We powered this trial to be able to detect a difference in GAF score of six points.

To detect a mean difference in global functioning of six points on the GAF (SD = 10.0) at 24 months with a two-sided significance level (α) of 5% and power of 80% would require 45 patients in each arm of the trial. In trials of complex interventions there is likely to be clustering of the intervention effect within therapists. In our recent trial of music therapy for people with schizophrenia we observed an intra-class correlation coefficient (ICC) of 0.125 [35]. However we anticipated that group processes may lead to a greater clustering of effects and decided to use an ICC of 0.175 for this trial. With an estimated cluster size of 8 and an ICC of 0.175 the Design Effect for the trial is 2.22 and a sample size of 100 per group was therefore required. A sample of 100 participants in each of the three arms of the trial would be sufficient to detect a difference of 50% in mean costs, at the 5% level of significance and with 80% power. In anticipation of a 20% loss to follow up at 24 months, we planned to randomise 376 participants, 94 at each centre.

### Statistical analysis

All primary statistical analysis will use the intention-to-treat principle. The statistical package STATA (version 11.0) will be used for all the analyses. The numbers (with percentages) of losses to follow-up at 12, and 24 months after randomisation will be reported and compared between the treatment arms with absolute risk differences (95% Confidence Intervals); any deaths and their causes will be reported separately.

For our main analysis we will impute baseline missing covariates using either mean or regression imputation to increase power and precision of the estimated treatment effect [36]. We will use all available results without imputation of missing outcomes. For the continuous outcomes, differences in mean score between those randomised to each of the three arms of the trial will be examined using analysis of covariance adjusting by 1) site and baseline value of outcome 2) site, baseline value of the outcome, sex and age. The assumption of linearity will be assessed by residual analysis; if necessary bootstrapping techniques will be employed.

Two sensitivity analyses will be conducted to take into account missing data 1) multiple imputation, which assumes data are 'missing at random', and 2) replacement of the missing GAF scores with those from the GAF proxy measure that we collected from participants' key workers.

We anticipate that there will be clustering of outcomes as a result of patients being assigned to groups facilitated by different therapists in different sites. Such clustering violates the assumption that observed outcomes of individuals are independent and can result in increased standard errors [37,38]. To take account of this we will explore separately therapist and site as random effects and finally a three-level model will be fitted, with patients as level one, therapist as second level, and the site as the third level. If our conclusions depend on which model is adopted we will present all results in the principal paper.

In a secondary analysis we will examine the impact of the level of uptake of groups using Complier Average Causal Effect analysis [39]. Instrumental variable methods will be used to model our outcome adjusting for age and sex. Randomisation allocation will be used as an instrumental variable.

The health economic evaluation will be conducted from the societal perspective, covering services received and any productivity losses. Differences in mean costs will be analysed using standard parametric t-tests with the validity of results confirmed using bias-corrected, nonparametric bootstrapping (repeat re-sampling) [40]. Despite the skewed nature of cost data, this approach is recommended to enable inferences to be made about the arithmetic mean [41]. In a secondary analysis, cost-effectiveness will be assessed through the calculation of incremental cost-effectiveness ratios [42] and will be explored in terms of global functioning (primary analysis) and quality adjusted life years using the EQ-5 D measure of health-related quality of life. Uncertainty around the cost and effectiveness estimates will be represented by cost-effectiveness acceptability curves [43].

A full Statistical Analysis Plan was developed by the team and ratified by an independent Trial Steering Group prior to data analysis.

## Discussion

The MATISSE trial provides the first opportunity to examine the effects and cost effectiveness of group Art Therapy compared to an active control treatment for people with schizophrenia. In comparing outcomes of those referred to group Art Therapy with those of people referred to an activity group, we will be able to compare levels of engagement with these different types of groups and to explore whether any benefit associated with group Art Therapy goes beyond that associated with referral to a less specialised group. By collecting follow-up data 24 months after randomisation we will also be able to examine any long term benefit associated with referral for group Art Therapy.

Since starting the trial national guidance on the treatment of schizophrenia in England has been published which recommend that clinicians should consider offering arts therapies to all people with schizophrenia, particularly for the alleviation of negative symptoms [19]. This recommendation is based on a synthesis of findings from exploratory trials of a range of different individual and group-based arts therapies. The MATISSE study provides an opportunity to examine the impact of an arts therapy when offered to a wider group of people with schizophrenia across a range of different clinical settings.

### Status of the trial

Recruitment to the study commenced in January 2007 and ended in September 2008. Four-hundred and seventeen participants were recruited and the final follow-up interviews are due to be completed by September 2010.

## Competing interests

The authors declare that they have no competing interests.

## Authors' contributions

The trial was initiated by MJC, DW and HK who, with SB, AM, KC, TRB, DO, TJ, MK and PT, designed the trial. EK took a lead in developing the data analysis plan. BB, SP, TS and FAO helped refine study methods and contributed to the collection and management of study data. All authors read and approved the final manuscript.

## Pre-publication history

The pre-publication history for this paper can be accessed here:

http://www.biomedcentral.com/1471-244X/10/65/prepub
